# Enhanced spinal neuronal responses as a mechanism for increased number and size of active acupoints in visceral hyperalgesia

**DOI:** 10.1038/s41598-020-67242-9

**Published:** 2020-06-25

**Authors:** Yu Fan, Yeonhee Ryu, Rongjie Zhao, Kyle B. Bills, Scott C. Steffensen, Chae Ha Yang, Hee Young Kim

**Affiliations:** 1 Department of Physiology, College of Korean Medicine, Daegu Haany University, Daegu, 42158 South Korea; 2 Clinical Medicine Division, Korea Institute of Oriental Medicine, Daejeon, 34054 South Korea; 3 Department of Psychopharmacology, Qiqihar Medical University, Qiqihar, 161006 China; 4 Department of Psychology and Neuroscience, Brigham Young University, Provo, Utah 84602 USA

**Keywords:** Physiology, Neurophysiology, Perception, Neuroscience, Somatosensory system, Central nervous system

## Abstract

Acupuncture has been used to treat a variety of illness and involves the insertion and manipulation of needles into specific points on the body (termed “acupoints”). It has been suggested that acupoints are not merely discrete, static points, but can be dynamically changed according to the pathological state of internal organs. We investigated in a rat model of mustard oil (MO)-induced visceral hyperalgesia whether the number and size of acupoints were modified according to the severity of the colonic pain, and whether the changes were associated with enhanced activity of the spinal dorsal horn. In MO-treated rats, acupoints showing neurogenic inflammation (termed “neurogenic spots” or Neuro-Sps) were found both bilaterally and unilaterally on the leg. The number and size of these acupoints increased along with increasing doses of MO. Electroacupuncture of the acupoints generated analgesic effects on MO-induced visceral hypersensitivity. The MO-treated rats showed an increase in c-Fos expression in spinal dorsal horn neurons and displayed increased evoked activity and a prolonged after-discharge in spinal wide dynamic response (WDR) neurons in response to colorectal distension. Increased number and size of neurogenic inflammatory acupoints following MO treatment were reduced by inhibiting AMPA and NMDA receptors in the spinal cord. Our findings suggest that acupoints demonstrate increased number and size along with severity of visceral pain, which may be associated with enhanced neuronal responses in spinal dorsal horn neurons.

## Introduction

Acupuncture, as an important intervention in Traditional Chinese Medicine (TCM), has been used to treat a variety of conditions, disorders, and diseases. Acupuncture points or “acupoints” are the specific sites on the body that are stimulated by the insertion of a thin needle or by the application of pressure to achieve therapeutic effects in TCM^1^. According to TCM theory, each acupoint communicates with a specific visceral organ and can reflect or treat the conditions of the organ^1,2^. In support of it, an acupoint becomes hypersensitive or tender under certain pathological conditions of visceral organs^3,4^. Manual or electrical stimulation of acupoints can relieve the symptoms of the associated visceral organs^5^, possibly via an endogenous opioid mechanism^6,7^.

It has been suggested that acupoints are not discrete, static points but are dynamically changing according to visceral disorders. The size and function of acupoints can be changed with the magnitude and status of the disorder. The process of dynamic changes in acupoints includes the transformation on the body surface from a silent mode under healthy conditions to an activated or sensitized mode under pathological conditions. The therapeutic effects of acupuncture on the corresponding visceral organ can be generated while acupoints are in activated or sensitized states^8^. Until now, this concept has not been proven due to the limitations in the scientific approach to studying acupoints. On the other hand, our previous study showed experimental evidence that acupoints can be identified as cutaneous “neurogenic spots” or Neuro-Sps, which are characterized by plasma extravasation and vasodilation in the postcapillary venules of the skin arising from release of calcitonin gene-related peptide (CGRP) and substance P (SP) from activated small diameter sensory afferents, and can be visualized experimentally in the skin by systemic injection of Evans blue dye (EBD)^5^. By using the EBD technique to visualize acupoints, it may be possible to evaluate the TCM concept (“dynamic changes of acupoints”) that number and size of acupoints change dynamically with visceral conditions.

Visceral pain can produce referred pain at topographically distinct areas of the body due to the convergence of visceral and somatic afferents on the same neuron in the sensory pathway^9^. In multiple sites of skin overlying the referred pain, local tissue responses are often accompanied by tenderness and hyperalgesia. Visceral pain can also trigger a central amplification termed “sensitization”, which refers to the amplification of neural signaling within the central nervous system that elicits pain hypersensitivity^10^. The central sensitization from visceral nociceptive afferents facilitates the somatic afferent and enhances skin sensitization and hyperalgesia on body surfaces, which may result in the increase of tender points or sensitive points on the skin^11^. In addition, the expansion of the peripheral receptive field by sensitization of spinal dorsal horn neurons leads to a wider and more intense transmission of nociceptive information^10^, which may be associated with increased size of sensitized points in skin. Thus, we hypothesized that enhanced neuronal responses (central sensitization) in the spinal cord may be associated with the dynamic changes of acupoints in TCM.

By using a rat model of colorectal inflammation produced by an irritant mustard oil (MO) and colorectal distension (CRD), the present study investigated whether: (1) The number and size of disease-associated acupoints are changed according to the severity of the colonic pain; (2) The electrical stimulation of these acupoints generates therapeutic effects on colonic pain; (3) The number and size of acupoints are associated with enhanced responses of spinal dorsal horn neurons; and (4) The effect is blocked by AMPA and NMDA receptor antagonists.

## Materials and Methods

### Animals

Adult male Sprague-Dawley rats (Hyochang, Seoul, Korea) weighing 250–350 g were used. Animals were housed at constant humidity (40~60%) and temperature (22 ± 2 °C) with a 12-hour light/dark cycle and allowed free access to food and water. All procedures were carried out in accordance with the National Institutes of Health Guide for the Care and Use of Laboratory Animals and approved by the Institutional Animal Care and Use Committee (IACUC) at Daegu Haany University. Rats were deprived of food, but not water, 12 hours before conducting experiments.

### Chemicals

Evans blue dye (EBD; 50 mg/ml in saline; Sigma, USA), mineral oil (vehicle for mustard oil^12,13^; Sigma), mustard oil (MO, Allyl isothiocyanate, 95%, Sigma). D-(-)−2-amino-5-phosphonopentanoic acid (D-AP5, 10 mM in saline, TOCRIS, UK; an NMDA receptor antagonist), and 6-Cyano-7-nitroquinoxaline-2, 3-dione (CNQX, 2 mM in saline, TOCRIS; an AMPA/kainate receptor antagonist).

### Induction of inflammatory visceral pain by mustard oil

The method of colorectal inflammation by MO was used as described previously^14^ with a slight modification. A lavage tube was inserted into colon and rectum through the anus at a depth 8 cm, and the different doses of MO (5, 10, 50 and 100 μl) or mineral oil (vehicle, 100 μl) were injected when needed.

### Detection of neurogenic inflammation in the skin by EBD injection

Neurogenic inflammatory spots (Neuro-Sps) were visualized by injecting EBD (50 mg/kg) intravenously (IV) in male Sprague-Dawley rats as described previously^5,15^. Ten min after infusion of MO or vehicle into the colon, the distal portion of the tail was dipped into 40 °C warm water for at least 30 sec to dilate the tail vein. As significant pain behaviors are observed within 10 min after colon instillation of MO^16^, EBD was injected 10 min after MO. EBD was then injected into the tail vein with a 26 gauge needle, and skin color changes were observed up to 1 hour after EBD injection. The number of blue-dyed Neuro-Sps on body surfaces were counted and the sizes of spots were measured by using a digital caliper (Mitutoyo, Japan). Neuro-Sps on the skin were sketched using body charts, photographed and compared with a human acupoint chart based on the transpositional method, which locates acupoints on the surface of animal skin corresponding to the anatomic site of human acupoints^17^.

### Colorectal distension

Colorectal distension (CRD) was accomplished using a 5 cm balloon attached to an intravenous line via a T-type channel which was connected to a sphygmomanometer and pressure transducer, as previously described^18^. The balloon was gently inserted into the descending colon and rectum through the anus to the depth of about 8 cm to avoid direct stimulation to anus and bowel wall. CRD stimulation (20, 40, 60 and 80 mmHg) epochs were achieved by inflation of the balloon through the sphygmomanometer with a duration of 20 sec. To prevent possible sensitization triggered by frequent stimulation of colorectum, the interval between two bouts of CRD stimulation was at least 3 min.

### Measurement of electromyographic response to colorectal distension

A pair of silver wires (Teflon, bare/coated diameter: 0.1 mm/ 0.13 mm, A-M Systems Inc., USA) were inserted into the external oblique abdominis muscle, buried for 10 mm of their length and separated by 10 mm for electromyography (EMG) recording. EMG recordings in response to CRD stimulation for 20 sec at strengths of 20, 40, 60, and 80 mmHg were performed. The EMG signals were amplified through a Bio AMP device (Model FE231, AD Instruments, USA), filtered (60–300 Hz) and digitized through a PowerLab 4/30 data acquisition system (AD Instruments). The EMG signal was rectified, averaged over 200 msec and quantified by calculating area under the curve (AUC) of the rectified EMG signal trace for the 20 sec CRD. After measurement of baseline, mineral oil (vehicle, 100 μl) or MO (5, 10, 50, 100 μl) was infused into the colon through a lavage needle, approximately 8 cm proximal to the anus, kept for 30 min and withdrawn.

### Evaluation of behavioral responses to CRD

Behavioral responses to CRD were assessed by measuring the abdominal withdrawal reflex (AWR) as described previously^19^. Five abdominal withdrawal reflex (AWR) scores (AWR0 to AWR4) were used to assess the animal response though visual observation to graded CRD (20, 40, 60, and 80 mmHg): AWR0: emotionally stable and no behavioral response to CRD; AWR1: brief head movement followed by immobility; AWR2: mild abdominal muscle contraction; AWR3: lifting the abdomen off the platform or flatting of abdomen; AWR4: body arching or lifting pelvic structures off the platform. AWR was recorded 30 min after infusion of vehicle (mineral oil) or 10 μl MO (95%). Electroacupuncture (EA; 2 Hz, 0.5 mA, 0.1 ms, triangular pulses) was applied to the needles for 10 min, and AWR was then recorded in response to CRD stimulation 20 min after the EA treatment. For AWR measurement, the rats were given CRD for 20 seconds every 3 min. The CRDs were repeated 3 times at each pressure level and averaged for analysis.

### Acupuncture at neurogenic spots (Neuro-Sps)

Under light isoflurane anesthesia (1.5% isoflurane in 100% oxygen), acupuncture needles (0.10 mm in diameter) were inserted 3 mm deep into core or edge of Neuro-Sps. For electroacupuncture treatment, a reference needle was inserted 2 mm away from the center of the Neuro-Sp, and electrical stimulation (2 Hz, 0.5 mA, 0.1 ms, triangular pulses) was applied to the needles for 10 min. Twenty min after EA, the EMG responses or behaviors in response to CRD stimulation were then measured.

### Immunohistochemistry of c-Fos in the spinal dorsal horn

As reported previously^20^, the spinal cord were extracted 1 hour after colon infusion of MO, and fixed in paraformaldehyde (PFA), cryo-protected, and cryo-sectioned at 30 μm thickness. The sections were washed in phosphate buffered saline (PBS) three times (10 min/wash) and incubated in a blocking solution for 1 hour at 4 °C, followed by an incubation with primary antibody for c-Fos (1:15000, rabbit anti-c-Fos antibody, Abcam, USA) overnight at 4 °C. After being washed again in PBS three times (10 min/wash), the sections were incubated with secondary antibody (1:500, Alexa Fluor 594 donkey anti-rabbit IgG antibody, Thermo Scientific, USA) for 2 hours at room temperature. The sections were cover-slipped with Vectashield Hard Set mounting medium for fluorescence (Vector, USA). Images were taken from 6 sections from each animal with a laser-scanning confocal microscope (LSM700, Carl Zeiss, Germany) and quantified by using ImageJ software (National Institute of Mental Health, USA).

### ***In vivo*** extracellular single-unit recordings of wide-dynamic-range (WDR) neurons in the spinal cord

After 12 hours of fasting, rats were anesthetized with an intraperitoneal injection of urethane (1.5 g/kg). The rat was placed in a stereotaxic apparatus and the body temperature was kept constant at 37 °C using a feedback-controlled DC heating pad. A laminectomy was performed at the lumbar spine to expose the L1–L3 segments of the spinal cord, the corresponding vertebrae were fixed in a rigid frame, and the spinal cord was bathed in a pool of mineral oil. As described previously^21^, extracellular recordings were performed on wide dynamic range (WDR) dorsal horn neurons (0.5–1.5 mm lateral to the midline and 500–1500 μm beneath the surface of spinal cord). Cells were searched at the L1 and L3 segments of the spinal cord using a carbon-filament glass microelectrode (0.4–1.2 MΩ, Carbostar-1, Kation Scientific, USA) mounted on an electronic micromanipulator. Spontaneous discharges were amplified and filtered at 10–0.1 kHz (ISO-80; World Precision Instruments, USA). Single-unit activity was discriminated, recorded, and analyzed using a CED 1401 Micro3 device and Spike2 software (Cambridge Electronic Design, UK). Wide-dynamic range dorsal horn neurons were recognized based on their responses to both innocuous (brush) and noxious (pinch) mechanical stimuli at sensitive areas near the leg. They were excited by both noxious and innocuous stimulation applied to their skin receptive fields. After a WDR neuron was confirmed, graded CRD stimulations were applied, and it was recorded in response to CRD in normal (12 cells from 6 rats) or MO-treated rats (12 cells from 6 rats).

### Intrathecal injection of D-AP5 and CNQX

A mixture of D-AP5 and CNQX was intrathecally injected by using a modified lumbar puncture technique^22^. Briefly, the spinal process of the sixth lumbar (L6) was palpated with the index finger, and a 27-gauge hypodermic needle (32 mm) connected to a 100-μl Hamilton syringe was inserted from the caudal end, 2–3 mm lateral to the L6 spinous process at a 45 angle to the vertebral column and was pushed slowly toward the cranioventral direction. When a sudden lateral tail movement was observed, drug or saline was injected slowly for 30 sec and the syringe was held in place for over 10 sec to prevent outflow of the drug.

### Statistical analysis

Statistical analysis and calculation of sample size were carried out using SigmaStat 3.5 software (Systat Software Inc., USA). All data are presented as the mean ± standard error of the mean (SEM) and analyzed by one or two-way measures analysis of variance (ANOVA) with Tukey post hoc tests or unpaired t-tests where appropriate. Statistical significance was considered at p < 0.05. Sample size of animals per treatment group was calculated to provide a power of 0.8 and an alpha = 0.05.

## Results

### Increased number and size of Neuro-Sps with the magnitude of visceral pain

**Figure 1:**
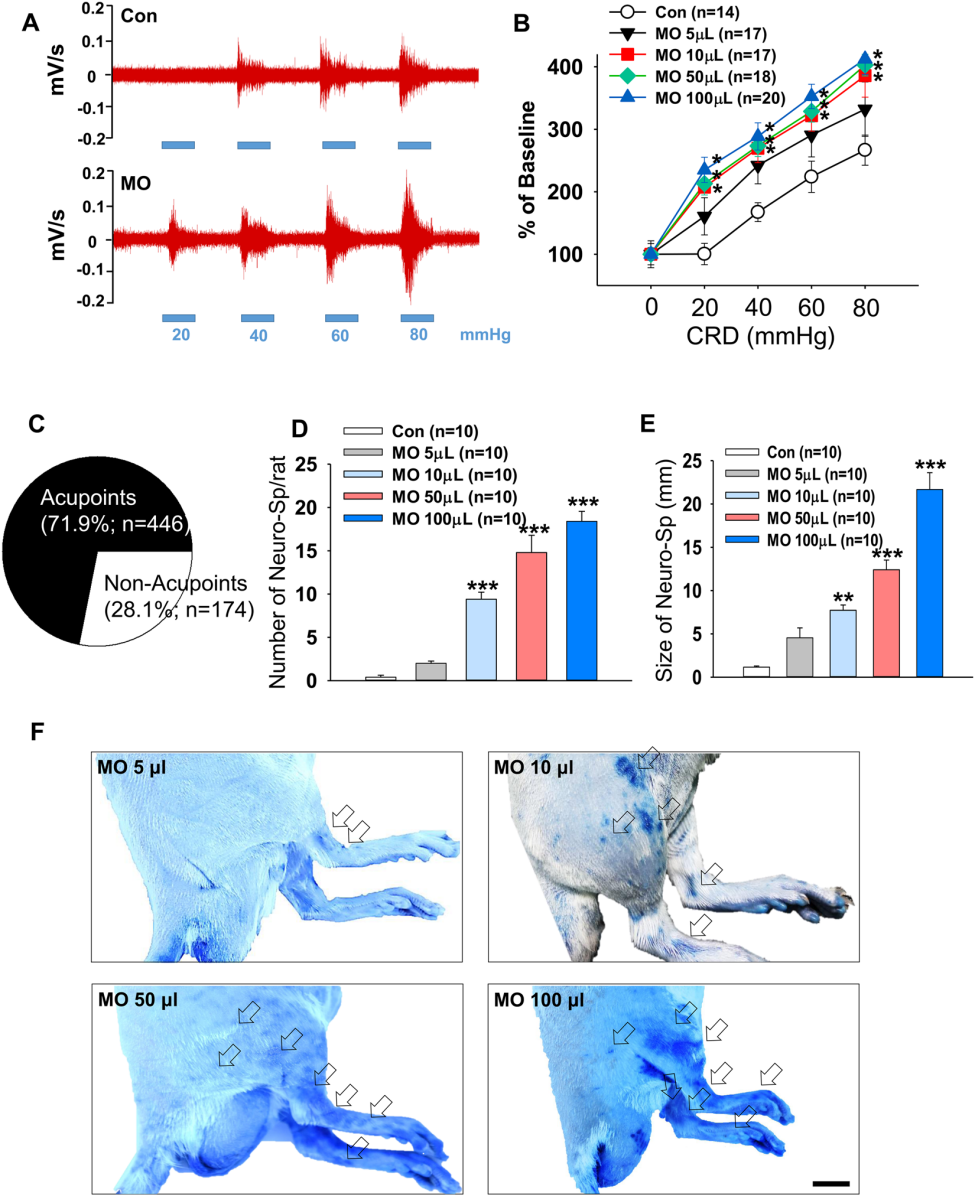
Increased number and size of Neuro-Sps along with severity of colonic pain in MO-treated rats. (**A**) Representative EMG activities in response to colorectal distension (CRD) in control or MO-infused rats. (**B**) EMG activities in the external oblique muscle in response to graded CRD in control or MO-infused rats. *p < 0.05 vs. Con. (**C**) Correlation of the anatomic location between Neuro-Sp and acupoints in MO-infused rats (n = 40). (**D**) Number of Neuro-Sps in MO-infused rats. ***p < 0.001 vs. Con. (**E**) Size of Neuro-Sps in MO-infused rats. **p < 0.01, ***p < 0.001 vs. Con. (**F**) Representative images of Neuro-Sps in MO-infused rats. Bar = 20 mm. MO, mustard oil; Con, Control. Arrows indicate prominent Neuro-Sps at each of MO.

To evaluate whether MO induces visceral hyperalgesia, four different volumes of MO (95%; 5, 10, 50 and 100 μl) were infused into the colon approximately 8 cm distant from anus and visceromotor reflex in response to colonic distension was measured. Colonic distension following MO infusion dose-dependently increased visceromotor reflex (two-way ANOVA, group F _(4,16)_ = 15.597, *p* < 0.001; dose F _(4,16)_ = 97.865, *p* < 0.001; interaction F _(16,405)_ = 1.063, *p* < 0.001; Fig. 1A,B).

Based on our previous findings demonstrating that the majority of acupoints correspond to Neuro-Sps on the skin^5^, we explored whether the acupoints match with Neuro-Sps in a rat model of visceral hyperalgesia. Approximately 10 min after the infiltration of MO into colon, Neuro-Sps were visualized by intravenous injection of EBD. The blue spots started to appear approximately 5 min after EBD injection. When Neuro-Sps in MO-treated rats (n = 40) were compared with the corresponding human anatomical acupoints, the majority appeared bilaterally or unilaterally on the leg, and 71% (446 of 620 spots; Fig. 1C) of the spots coincided with known acupoints of the lower limbs, the inside of lower leg and thigh (Fig. 1F), such as SP6, ST36, and BL60.

The rats treated with MO at 5, 10, 50 and 100 μl exhibited approximately 2, 9, 15, and 18 Neuro-Sps per rat, respectively, while vehicle-treated rats showed only a few spots (one-way ANOVA, F _(4, 45)_ = 50.248, *p* < 0.001; Fig. 1D). In addition, the rats that received MO showed significantly increased Neuro-Sp size (5 μl: 4.55 ± 1.14; 10 μl: 7.72 ± 0.62; 50 μl: 12.41 ± 1.12; 100 μl: 21.67 ± 1.94 mm), compared with 1.16 ± 0.13 mm in vehicle-treated control rats (Con; one-way ANOVA, F _(4, 260)_ = 37.57, *p* < 0.001; Fig. 1E). These results indicate that the majority of acupoints display cutaneous neurogenic inflammation and plasma extravasation in the rat model of MO-induced colonic pain and the number and size of these points increased along severity of the visceral pain. Since a significant increase in both visceral pain and Neuro-Sps was observed from 10 μl MO, the dosage of 10 μl MO was used in the following experiments.

### Stimulation of Neuro-Sps generated therapeutic effects on visceral pain

**Figure 2:**
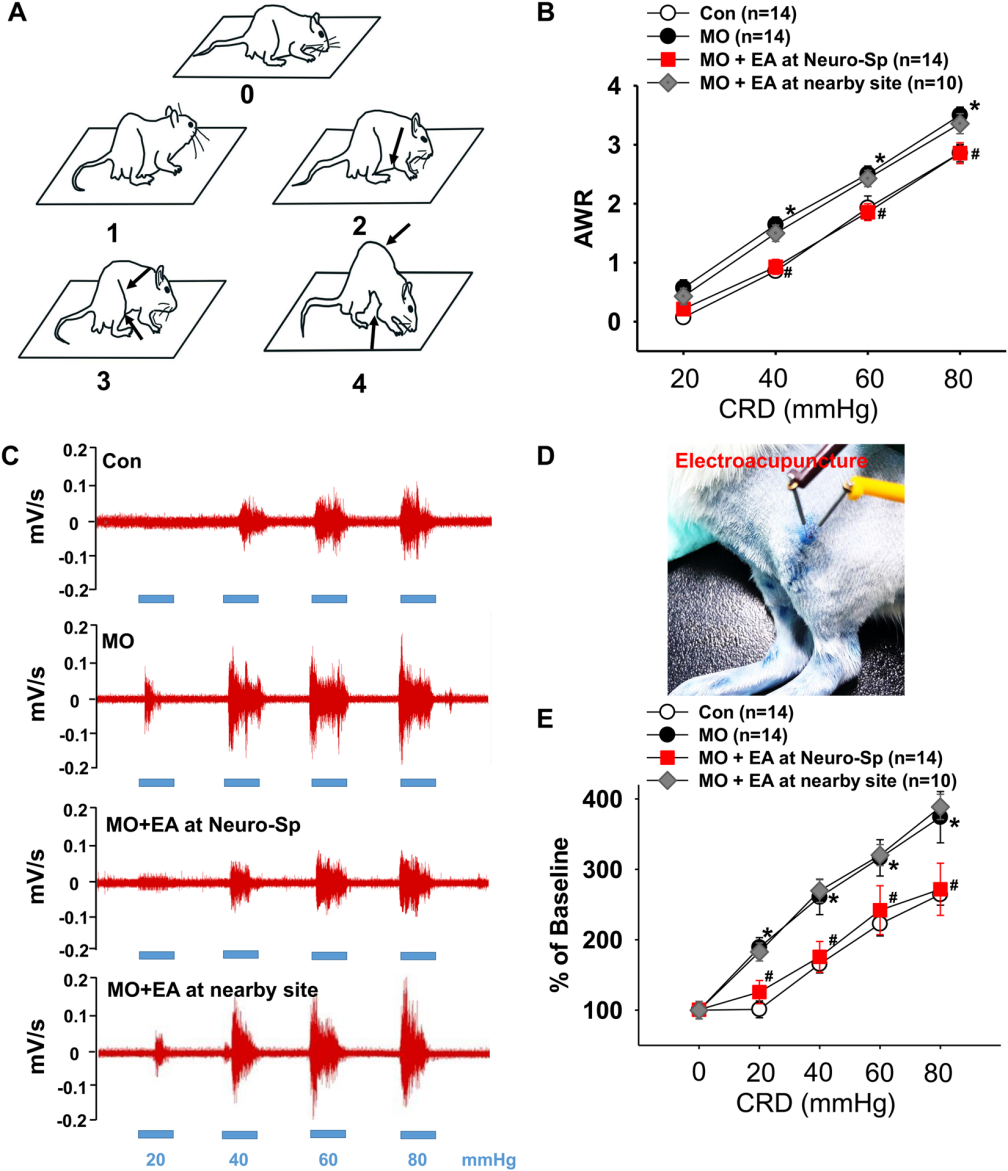
Visceral hypersensitivity and its reduction by electroacupuncture at Neuro-Sps. **(A**) Schematic drawings of rats on platforms illustrating the behavioral scale based on visual observations of the AWR in response to graded CRD. AWR, abdominal withdrawal reflex. (**B**) AWR scores induced by graded CRD in control rats or MO-infused rats or following electroacupuncture (EA) application in MO-infused rats (B). *p < 0.05 vs. Con; ^#^p < 0.05 vs. MO + EA at nearby site. (**C–E**) EMG activities in the external oblique muscle in response to graded CRD in control (Con) or MO-infused rats or following electroacupuncture application (D) in MO-infused rats. *p < 0.05 vs. Con; ^#^p < 0.05 vs. EA at nearby site.

**Figure 3:**
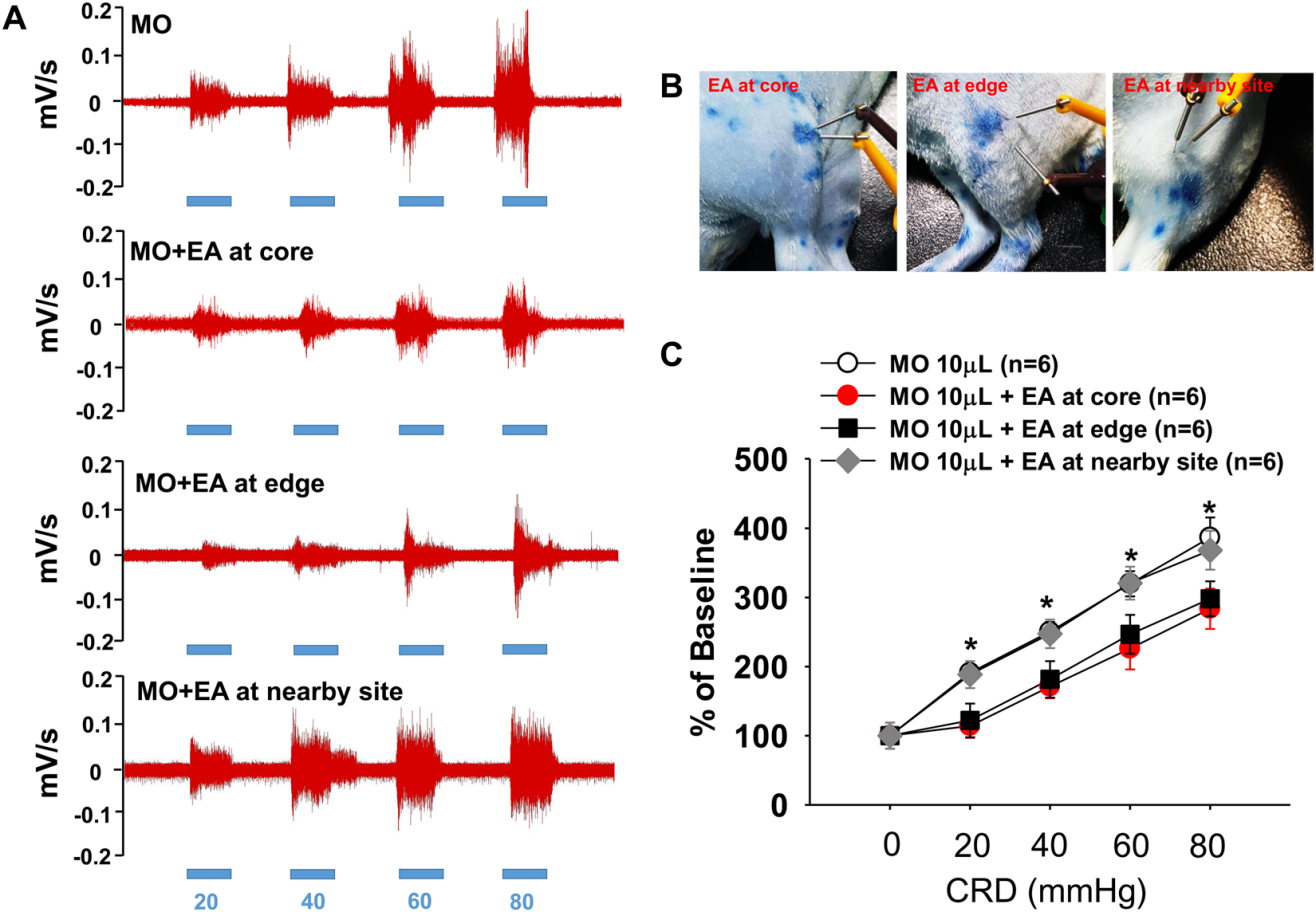
**A**–**C**) Effect of electroacupuncture at core, edge, or site outside (nearby site) of the Neuro-Sp on EMG activities in the external oblique muscle in response to graded CRD in MO-infused rats (MO) *p < 0.05 vs. MO. Representative EMG signals (**A**) and pictures for EA at core, edge or nearby site of Neuro-Sp (**B**).

We then determined whether the Neuro-Sps seen in MO-treated rats function as therapeutic acupoints. Two dominant Neuro-Sps on the knee area of hindlimb (Fig. 2D), were chosen for acupuncture stimulation in MO (10 μl)-treated rats. Enhanced AWR scores or EMG responses in response to graded CRD pressure were observed in MO-treated rats, compared with MO-treated controls. When electrical stimulation at hindlimb Neuro-Sps was applied for 10 min in MO-treated rats, it significantly reduced AWR scores and EMG activity in response to graded CRD, compared with controls (Con) or electroacupuncture (EA) at nearby sites (AWR: two-way ANOVA, group F _(3,9)_ = 20.704, *p* < 0.001; intensity F _(3,9)_ = 300.547, *p* < 0.001; interaction F _(9,208)_ = 0.391, *p* = 0.938; Fig. 2A,B; EMG: two-way ANOVA, group F _(3,12)_ = 26.528, *p* < 0.001; intensity F _(4,12)_ = 77.63, *p* < 0.001; interaction F _(12,260)_ = 1.854, *p* = 0.04; Fig. 2C,E). To further evaluate whether the entire area of the Neuro-Sp serves as an active acupoint, the effect of electrical stimulation at core, edge, or outside the Neuro-Sp on EMG activity in response to graded CRD was explored in MO (10 μl)-treated rats. Electrical stimulation at either core or edge of Neuro-Sps generated analgesic effects on visceral pain, compared with MO or EA at sites outside (nearby site) of the Neuro-Sp (two-way ANOVA, group F _(3,12)_ = 20.161, *p* < 0.001; intensity F _(4,12)_ = 105.566, *p* < 0.001; interaction F _(12,99)_ = 1.343, *p* = 0.212; Fig. 3A–C). These data showed that MO-treated rats were more sensitive to CRD than control rats and electroacupuncture at Neuro-Sps generated therapeutic effects like acupoints.

### Increased number and size of Neuro-Sps are associated with enhanced responses of spinal dorsal horn neurons

**Figure 4:**
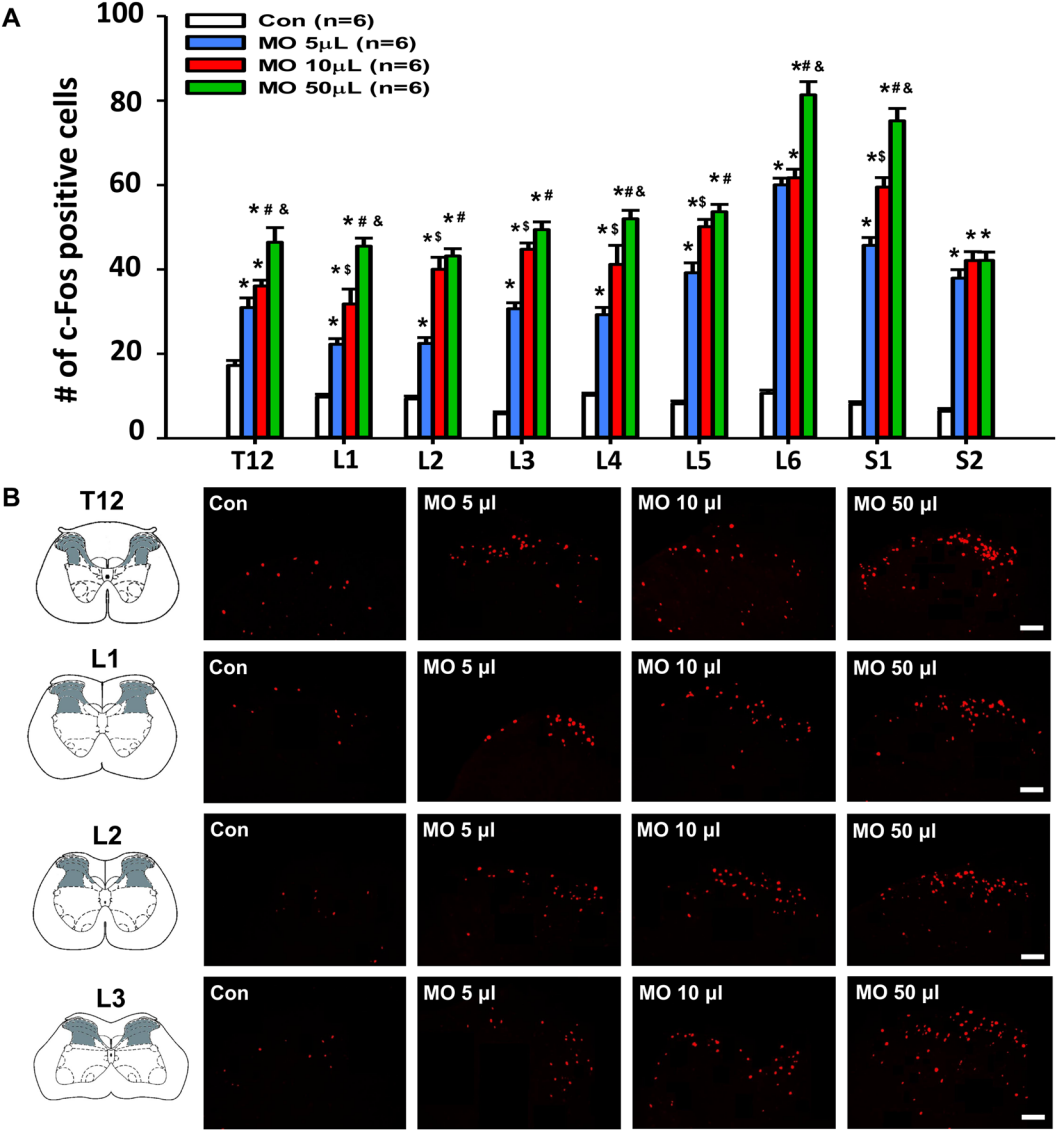
Increased c-Fos expression in the spinal dorsal horn following MO-induced colonic pain. **(A**) Quantification of c-Fos labeled neurons in the T12-S2 in MO-infused rats (n = 6/group). Data are expressed as the numbers of c-Fos-positive cells in the T12-S2 per mm^2^. *p < 0.05, MO 5 μl vs. Con (control); ^$^p < 0.05, MO 5 μl vs. MO 10 μl; ^#^p < 0.05, MO 5 μl vs. MO 50 μl; ^&^p < 0.05, MO 50 μl vs. MO 10 μl. (**B**) Representative images of c-Fos expression in the T12-L3 of the spine following Con (control; mineral oil) or MO 5, 10 or 50 μl application. Bar = 50 μm.

To determine if increased number and size of Neuro-Sps are associated with the extent of activation of spinal dorsal horn neurons, the expression of c-Fos was examined in spinal dorsal horns following different doses of MO. MO-treated rats (5, 10 or 50 μl, n = 6/group) showed a significant increase in c-Fos expression of dorsal horn in T12-S2 of spinal cord from that of vehicle-treated control rats (Con, n = 6; one-way ANOVA, T12: F _(3, 139)_ = 38.664, *p* < 0.001; L1: F _(3, 129)_ = 73.345, *p* < 0.001; L2: F _(3, 112)_ = 92.263, *p* < 0.001; L3: F _(3, 112)_ = 181.474, *p* < 0.001; L4: F _(3, 111)_ = 72.219, *p* < 0.001; L5: F _(3, 97)_ = 180.692, *p* < 0.001; L6: F _(3, 80)_ = 184.644, *p* < 0.001; S1: F _(3, 89)_ = 171.692, *p* < 0.001; S2: F _(3, 94)_ = 95.383, *p* < 0.001; Fig. 4A,B). Moreover, there were significant increases in the number of c-Fos positive cells following MO treatment in a dose-responsive manner.

**Figure 5:**
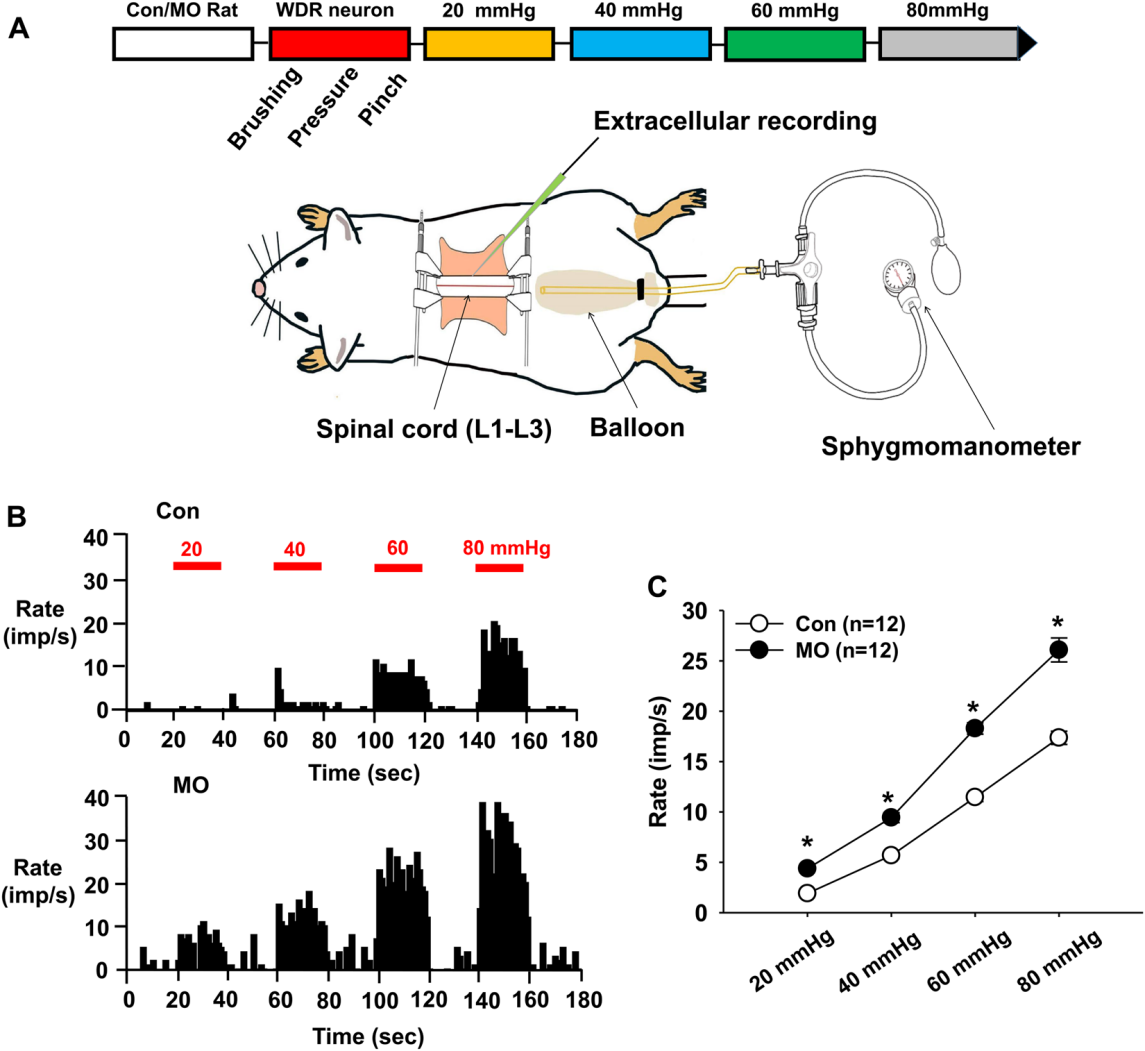
Enhancement of spinal WDR neuronal activities in MO-infused rats.**(A**) Experimental schedule (upper) and schematic illustration for extracellular recording in the spinal dorsal horn (lower). (**B,C**) Single-unit activity of WDR neurons in response to CRD in normal (n = 12) or MO-infused rats (n = 12). Representative histograms of neuronal responses to CRD (**B**). Summary of WDR neuronal responses to CRD (**C**). *p < 0.05 vs. Con (control). MO, mustard oil.

To further confirm enhanced responses of spinal dorsal horn neurons, we recorded 24 WDR neurons in vehicle or MO-treated rats (Con: n = 6; MO: n = 6) and measured the discharge rates induced by CRD stimulation at 20, 40, 60, and 80 mm Hg. In the control group, the mean activity of the WDR neurons was 1.91 ± 0.03, 5.68 ± 0.28, 11.45 ± 0.42, and 17.37 ± 0.65 spikes/sec in response to CRD of 20, 40, 60, and 80 mm Hg, respectively. On the other hand, MO-infused rats showed significantly increased evoked activity and a prolonged after-discharge in response to CRD of 20, 40, 60, and 80 mmHg (20 mmHg: 4.38 ± 0.18; 40 mmHg: 9.43 ± 0.49; 60 mmHg: 18.29 ± 0.56; 80 mmHg: 26.08 ± 1.19 spikes/sec, respectively), compared to that of the control group (two-way ANOVA, group F _(1,3)_ = 174.801, *p* < 0.001; intensity F _(3,3)_ = 394.971, *p* < 0.001; interaction F _(3,152)_ = 11.989, *p* < 0.001; Fig. 5A–C). The data revealed that spinal dorsal horn neurons were sensitized by infiltration of MO into colon, which may be associated with increased number and size of acupoints in MO-treated rats.

### Occurrence of Neuro-Sps was inhibited by blocking spinal NMDA/AMPA receptors

**Figure 6:**
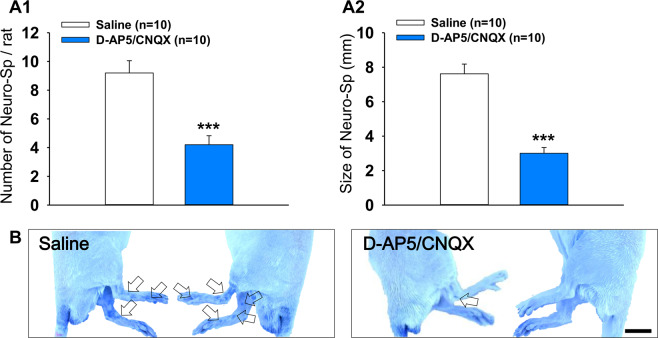
Reduction in Neuro-Sps by intrathecal D-AP5/CNQX in MO-infused rats. (**A**) Number (A1) and size (A2) of Neuro-Sps (n = 10) following an intrathecal injection of D-AP5/CNQX in MO-infused rats. ***p < 0.001 vs. Saline. (**B**) Representative images of Neuro-Sps comparing saline control to D-AP5/CNQX treatment. Bar = 20 mm.

To investigate whether inhibiting glutamatergic transmission in the spinal cord might reduce the number or size of Neuro-Sps, a mixture of D-AP5 and CNQX was intrathecally injected 10 min prior to colon infusion of 10 μl MO in rats. Intrathecal injection of D-AP5/CNQX reduced the number and size of Neuro-Sps (approximately 4.2 spots, 7.62 ± 0.57 mm; *p* < 0.001), compared with saline-treated rats (approximately 9.2 spots, 3.01 ± 0.33 mm; *p* < 0.001; Fig. 6A,B). The result showed that increased number and size of Neuro-Sps were associated with spinal NMDA/AMPA receptors.

## Discussion

The present study revealed that the number and size of neurogenic inflammatory acupoints (Neuro-Sps) increased along with severity of colonic pain in MO-treated rats. Acupuncture stimulation of the enlarged points suppressed the colonic pain. MO treatment increased c-Fos expression of dorsal horn T12-S2 in a dose-dependent manner. The discharge and after-discharge rates of spinal dorsal horn wide dynamic response (WDR) neurons in responses to CRD were enhanced in MO-treated rats, compared to those in normal rats. Moreover, the number and size of Neuro-Sps were reduced by intrathecal injection of D-AP5 and CNQX. Therefore, our findings suggest that the number and size of Neuro-Sps increased according to severity of visceral pain, which is associated with central sensitization in the spinal cord.

Some researchers have proposed that the size and function of acupoints can be changed according to the pathological state of internal organs^3,23^. When visceral function changes, the function of acupoint may be changed from “silenced status” into the “activated status”^8^. In support of this, the present study using behavioral, physiological, and molecular approaches showed that the number and size of Neuro-Sps increased along with severity of colonic pain in MO-treated rats. In our previous study, known acupoints display increased fluorescent intensity of CGRP and substance P, markedly dilated microvessels in dermis of Neuro-Sps and plasma extravasation^5,15^, suggesting activation or sensitization of acupoints under pathological visceral conditions.

We have proposed that Neuro-Sps correspond to traditional acupoints^5,24^. Visceral pain causes local skin release of neuropeptides CGRP and SP from activated small diameter sensory afferents and thus generates Neuro-Sps. The increased CGRP and SP in skin induce high mechanical sensitivity and plasma extravasation and enhanced electrical conductance. When small afferent fibers in Neuro-Sps are activated, needling sensation (Deqi) and therapeutic effects via endogenous opioid system can be produced^25–27^. The Neuro-Sps on skin are also connected to the internal organs. The features of Neuro-Sps overlap those of acupoints^5,24^. Consistent with previous study, the present study showed that the majority of Neuro-Sps (446 of 620 spots) matched with the acupoints on the hindlimb. We also showed that electroacupuncture (EA) stimulation of Neuro-Sps produced therapeutic effects by alleviating MO-induced colonic pain. Our previous study showed that electrical stimulation of Neuro-Sps alleviates pathological conditions in rat models of colitis and hypertension via the endogenous opioid system^5,24,28^, supporting the hypothesis that Neuro-Sps function as therapeutic acupoints.

In the present study, colon infusion of MO increased number and size of Neuro-Spsin a dose-dependent manner. Mustard oil treatment enhanced discharge rates of spinal dorsal horn WDR neurons in response to graded CRD stimuli. In addition, after-discharges following CRD stimuli were found in MO-treated rats, compared to controls, suggesting that colon MO infusion induces central sensitization and thus visceral hypersensitivity^29–31^, which results in increased number and size of Neuro-Sps. Consistent with our study, previous studies have revealed that MO-treated rats showed chronic visceral hypersensitivity, characterized by increased visceromotor response and enhanced responses of spinal dorsal neurons in response to colorectal distension. These rats also displayed mechanical hypersensitivity and increased responses of the spinal dorsal horn neurons to external stimuli, indicating both visceral hyperalgesia and enhanced somatic nociception. Therefore, the colonic irritation with MO results in somatic and visceral hypersensitivity^19^. In our previous study, Neuro-Sps displayed mechanical hypersensitivity^5^, suggesting that hyperexcitability of spinal dorsal horn neurons leading to visceral and somatic hypersensitivity is associated with increased size of Neuro-Sps.

Consistent with our previous study, irritant-treated rats revealed Neuro-Sps mainly at the hindpaw and frequently in the tail, thighs or lower back, which corresponded to spinal cord segments L2-S2^24,32^. In the present study, significantly increased c-Fos expression was found from T12 through S2. Since the colon is innervated by T12-L2 and L6-S1 spinal cord segments and those of hindpaw mainly enter to L4-L5 spinal segments, no overlapping spinal segments are observed between hindpaw and colon. However, during an inflammatory state of the colon the spinal neurons in nearby segments, including L4-L5, can be activated, providing a neuroanatomical correspondence for Neuro-Sps^33^, suggesting that an increase in spinal distribution of c-Fos expression following MO treatment would expand the ranges of dermatome including T12-S2 and lead to the numbers of Neuro-Sps in a dose-dependent manner.

Intrathecal injection of AMPA/NMDA receptor antagonists decreased both the number and size of Neuro-Sps following MO treatment. AMPA and NMDA receptors play important roles in the development of central sensitization and thus visceral hypersensitivity in animal pain models^34^. Previous studies showed that colonic infusion of MO induces acute colonic pain and results in visceral hypersensitivity and central sensitization^8,35–37^. Moreover, previous studies also found that NMDA and AMPA receptors are involved in the induction of c-Fos expression in the spinal cord evoked by MO activation of C-fibers^38^ and activation of dorsal horn neurons following application of mustard oil^39,40^. Blocking AMPA/NMDA receptors would inhibit central sensitization and visceral/cutaneous hypersensitivity, thereby leading to a decrease of Neuro-Sp acupoints in skin.

A limitation of this study is that the concentrations of Evans blue dye used can’t be applied to humans due to a potential risk of adverse events. However, it is possible that a much smaller concentrations (as low as 5.19 µg/kg) could be injected and still be fluorescently visualized^41^. This dosage is below that which has been used for decades to estimate blood volume in humans^42^. Regardless, Neuro-Sps can be identified by non-invasive methods, such as von Frey methods, infrared thermal imaging or electrodermal measurement, as shown in our previous studies^5,15^. Use of these non-invasive methods may aid in the visualization, diagnosis, and treatment of neurogenic inflammation.

In conclusion, colonic pain activates the viscerosomatic convergent neurons in the sensory pathway, and the neurons antidromically activate the branches, leading to the release of neuropeptides CGRP and SP from small diameter sensory fibers and subsequent sensitization of primary afferent nociceptors^43^, and generation of Neuro-Sps^11,44^. The number and size of Neuro-Sps increased along with severity of colonic pain in MO-treated rats, which was associated with enhanced activation of spinal AMPA/NMDA receptors. Stimulation of Neuro-Sps decreased colonic pain, possibly by recruiting endogenous opioid mechanism^5^.

## References

[1] Stux, G. & Pomeranz, B. *Acupuncture: textbook and atlas*. (Springer Science & Business Media, 2012).

[2] Rong P (2011). Mechanism of acupuncture regulating visceral sensation and mobility. Frontiers of medicine.

[3] Ben H (2012). Observation of Pain-Sensitive Points along the Meridians in Patients with Gastric Ulcer or Gastritis. Evid Based Complement Alternat Med.

[4] Chae Y (2007). The alteration of pain sensitivity at disease-specific acupuncture points in premenstrual syndrome. J Physiol Sci.

[5] Kim DH (2017). Acupuncture points can be identified as cutaneous neurogenic inflammatory spots.

[6] Han JS (2003). Acupuncture: neuropeptide release produced by electrical stimulation of different frequencies. Trends in neurosciences.

[7] Takahashi, T. Mechanism of acupuncture on neuromodulation in the gut–a review. *Neuromodulation: journal of the International Neuromodulation Society* **14**, 8–12; discussion 12, 10.1111/j.1525-1403.2010.00295.x (2011). 10.1111/j.1525-1403.2010.00295.x 21992155

[8] Rong PJ (2013). Peripheral and spinal mechanisms of acupoint sensitization phenomenon. Evid Based Complement Alternat Med.

[9] Procacci P, Maresca M (1999). Referred pain from somatic and visceral structures. Current Review of Pain.

[10] Woolf CJ (2011). Central sensitization: implications for the diagnosis and treatment of pain. Pain.

[11] Wesselmann U, Lai J (1997). Mechanisms of referred visceral pain: uterine inflammation in the adult virgin rat results in neurogenic plasma extravasation in the skin. Pain.

[12] Lu CL (2007). Estrogen rapidly modulates mustard oil-induced visceral hypersensitivity in conscious female rats: A role of CREB phosphorylation in spinal dorsal horn neurons. Am J Physiol Gastrointest Liver Physiol.

[13] Wu J (2007). The role of c-AMP-dependent protein kinase in spinal cord and post synaptic dorsal column neurons in a rat model of visceral pain. Neurochem Int.

[14] Peng HY (2009). Colon mustard oil instillation induced cross-organ reflex sensitization on the pelvic-urethra reflex activity in rats. Pain.

[15] Fan Y (2018). Neuropeptides SP and CGRP Underlie the Electrical Properties of Acupoints. Front Neurosci.

[16] Laird JM, Souslova V, Wood JN, Cervero F (2002). Deficits in visceral pain and referred hyperalgesia in Nav1.8 (SNS/PN3)-null mice. J Neurosci.

[17] Yin CS (2008). A proposed transpositional acupoint system in a mouse and rat model. Res Vet Sci.

[18] O’Mahony, S. M., Tramullas, M., Fitzgerald, P. & Cryan, J. F. Rodent models of colorectal distension. *Curr Protoc Neurosci* Chapter **9**(Unit 9), 40, 10.1002/0471142301.ns0940s61 (2012). 10.1002/0471142301.ns0940s61 23093353

[19] Al-Chaer ED, Kawasaki M, Pasricha PJ (2000). A new model of chronic visceral hypersensitivity in adult rats induced by colon irritation during postnatal development. Gastroenterology.

[20] Lu Y, Westlund KN (2001). Effects of baclofen on colon inflammation-induced Fos, CGRP and SP expression in spinal cord and brainstem. Brain Res.

[21] Yu, L. *et al*. *Inhibition of electroacupuncture on nociceptive responses of dorsal horn neurons evoked by noxious colorectal distention in an intensity-dependent manner*. Volume 12 (2019). 10.2147/JPR.S182876 PMC6322705 30655692

[22] Mestre C, Pelissier T, Fialip J, Wilcox G, Eschalier A (1994). A method to perform direct transcutaneous intrathecal injection in rats. J Pharmacol Toxicol Methods.

[23] Li Y-Q, Zhu B, Rong P-J, Ben H, Li Y-H (2006). Effective regularity in modulation on gastric motility induced by different acupoint stimulation. World journal of gastroenterology.

[24] Kim HY (2006). Skin on GV01 acupoint in colonic inflammatory states: tenderness and neurogenic inflammation. J Physiol Sci.

[25] Schoen A (2001). Veterinary Acupuncture. Mosby Ch..

[26] Hui KK (2007). Characterization of the “deqi” response in acupuncture. BMC complementary and alternative medicine.

[27] Pomeranz, B. & Berman, B. In *Basics of Acupuncture* (eds Gabriel Stux, Brian Berman, & Bruce Pomeranz) 7-86 (Springer Berlin Heidelberg, 2003).

[28] Kim HY (2005). Effects of acupuncture at GV01 on experimentally induced colitis in rats: possible involvement of the opioid system. Jpn J Physiol.

[29] Lu C-L (2007). Estrogen rapidly modulates mustard oil-induced visceral hypersensitivity in conscious female rats: a role of CREB phosphorylation in spinal dorsal horn neurons. American Journal of Physiology-Gastrointestinal and Liver Physiology.

[30] McMahon SB, Abel C (1987). A model for the study of visceral pain states: chronic inflammation of the chronic decerebrate rat urinary bladder by irritant chemicals. Pain.

[31] Jiang MC, Gebhart GF (1998). Development of mustard oil-induced hyperalgesia in rats. Pain.

[32] Takahashi Y, Nakajima Y (1996). Dermatomes in the rat limbs as determined by antidromic stimulation of sensory C-fibers in spinal nerves. Pain.

[33] Honore P, Kamp EH, Rogers SD, Gebhart GF, Mantyh PW (2002). Activation of lamina I spinal cord neurons that express the substance P receptor in visceral nociception and hyperalgesia. The journal of pain: official journal of the American Pain Society.

[34] Latremoliere A, Woolf CJ (2009). Central sensitization: a generator of pain hypersensitivity by central neural plasticity. J Pain.

[35] Kimball ES, Palmer JM, D’Andrea MR, Hornby PJ, Wade PR (2005). Acute colitis induction by oil of mustard results in later development of an IBS-like accelerated upper GI transit in mice. Am J Physiol Gastrointest Liver Physiol.

[36] Urban MO, Gebhart GF (1999). Supraspinal contributions to hyperalgesia. Proc Natl Acad Sci USA.

[37] Woolf CJ, Shortland P, Sivilotti LG (1994). Sensitization of high mechanothreshold superficial dorsal horn and flexor motor neurones following chemosensitive primary afferent activation. Pain.

[38] Soyguder Z (2004). NMDA and AMPA/KA receptors are involved in the c-Fos expression following mustard oil activation of C-fibres. J Chem Neuroanat.

[39] Woolf CJ, King AE (1990). Dynamic alterations in the cutaneous mechanoreceptive fields of dorsal horn neurons in the rat spinal cord. J Neurosci.

[40] Young MR, Fleetwood-Walker SM, Mitchell R, Dickinson T (1995). The involvement of metabotropic glutamate receptors and their intracellular signalling pathways in sustained nociceptive transmission in rat dorsal horn neurons. Neuropharmacology.

[41] Ryu H-W (2018). Low-Dose Evans Blue Dye for Near-Infrared Fluorescence Imaging in Photothrombotic Stroke Model. Int J Med Sci.

[42] Gregersen MI (1944). A practical method for the determination of blood volume with the dye T-1824. Journal of Laboratory and Clinical Medicine.

[43] Sahbaie P (2009). Role of substance P signaling in enhanced nociceptive sensitization and local cytokine production after incision. Pain.

[44] Arendt-Nielsen L (2008). Viscero-somatic reflexes in referred pain areas evoked by capsaicin stimulation of the human gut. European journal of pain (London, England).

